# p16 in HPV-associated cancers

**DOI:** 10.18632/oncotarget.1523

**Published:** 2013-10-31

**Authors:** Karl Munger, Tyshia K. Gwin, Margaret McLaughlin-Drubin

**Affiliations:** Division of Infectious Diseases, Brigham and Women's Hospital, Department of Medicine and Program in Virology, Harvard Medical School, Boston, MA USA; Division of Infectious Diseases, Brigham and Women's Hospital, Department of Medicine and Program in Virology, Harvard Medical School, Boston, MA USA; Division of Infectious Diseases, Brigham and Women's Hospital and Department of Medicine, Harvard Medical School, Boston, MA, USA

Infections with high-risk human papillomaviruses (HPV) account for approximately 5% of all human cancers. Cervical cancer, in particular, is almost universally caused by “high-risk” HPV infections and these HPVs also cause a large fraction of other anogenital tract cancers as well as oral carcinomas. The viral E6 and E7 proteins are the universal oncogenic drivers of HPV-associated cancers, and persistent E6/E7 expression is necessary for the maintenance of the transformed state. Hence the HPV E6 and E7 proteins should represent excellent therapeutic targets. The HPV E6 and E7 proteins lack intrinsic enzymatic activities and attempts to discover small molecule inhibitors of E6 or E7 containing cellular protein complexes have been futile. HPV E6 and E7 function by associating with host cellular proteins, thereby functionally re-programming cellular signal transduction pathways. The resulting rewiring of cellular circuitry causes “addictions” to certain signaling pathways that are non-essential and/or redundant in normal cells. This in turn generates specific cellular vulnerabilities that may be more readily druggable.

**Figure 1:**
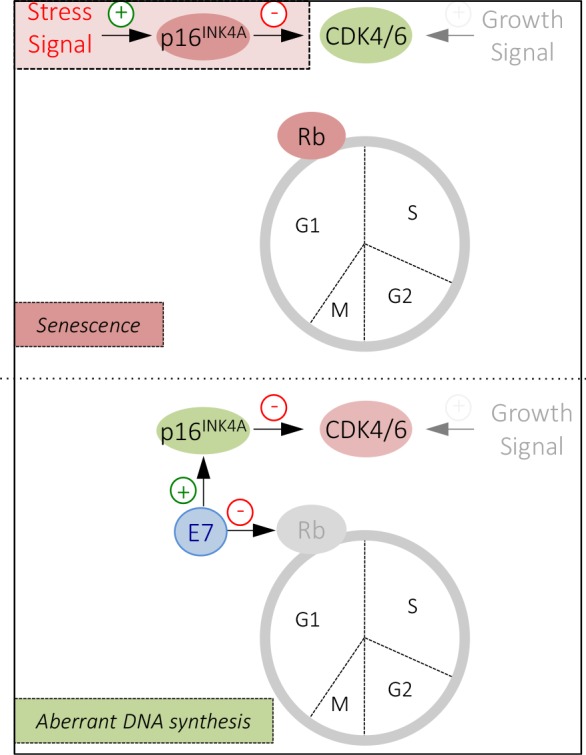
The retinoblastoma (Rb) tumor suppressor pathway p16^INK4A^ inhibits CDK4/6 This overrides growth signals and results in G1 growth arrest and senescence (upper panel). The high-risk HPV E7 proteins, which trigger p16^INK4A^ expression, have evolved to overcome this response by targeting Rb for degradation. Under these conditions, p16^INK4A^ expression and CDK4/6 inhibition is required for cell survival. Hence p16^INK4A^, which normally functions as a tumor suppressor (red) now has oncogenic activities (green) and similarly, CDK4/6, which normally are oncogenic (green) are now tumor suppressive (red), demonstrating the biological activities of key components of the retinoblastoma tumor suppressor pathway are dependent on the cellular context (see text for details).

The article (McLaughlin-Drubin ME et al., Proc. Natl. Acad. Sci. USA. 2013; 110:16175-16180) identifies a surprising and potentially druggable vulnerability in the retinoblastoma tumor suppressor pathway caused by the high-risk HPV E7 protein. High-risk HPV E7 protein expression triggers a cellular defense response referred to as oncogene induced senescence (OIS), which is mediated by the p16^INK4A^ (p16) tumor suppressor and executed by the retinoblastoma tumor suppressor protein, Rb. In normal cells, p16 expression is epigenetically silenced by polycomb repressive complexes. p16 inhibits cyclin dependent kinase 4 and 6 (CDK4/6). This causes accumulation of hypophosphorylated Rb, which in turn signals cell cycle arrest (Fig. 1 upper panel). E7 protein triggers p16. High-level p16 expression is an excellent biomarker for high-risk HPV-associated lesions and cancers. Cell cycle arrest and senescence in these tumors, however, is subverted by E7 targeting Rb for proteasomal degradation (Fig. 1 lower panel). p16 induction, in the absence of Rb (through Rb degradation by E7) causes a unique and surprising addiction to p16^INK4A^ expression. Even though p16 is a tumor suppressor in most cellular contexts, it is essential for survival of cervical carcinoma lines. The iconoclastic “oncogenic” activity of p16 in HPV E7 expressing cells, like its more familiar tumor suppressor activity, is based on the ability to inhibit CDK4/6. Indeed, a human melanoma derived p16-insensitive CDK4 mutant also causes cell death when expressed in HPV E7-expressing cells. Hence, this oncogenic CDK4 mutant exhibits biological activities that are akin to those of a tumor suppressor in the context of a p16-expressing, Rb defective cell. These results imply that CDK4/6 activity is not tolerated in cells that lack Rb activity. One may hypothesize that there must be CDK4/6 substrates that cause cell death when they are phosphorylated in cells that lack Rb. Tumorigenic activities of the Rb pathway including CDK4/6 and p16 are context dependent. CDK4/CDK6 inhibition may be efficacious only in tumors that retain functional Rb. In contrast, CDK4/CDK6 inhibition in cancers that have suffered Rb mutations is necessary for tumor growth and survival. In such cases it may be therapeutically useful to activate rather than to inhibit CDK4/CDK6 activity. One way CDK4/6 activation may be achieved is by epigenetic silencing of p16 through inhibition of the KDM6B enzyme. Indeed the KDM6-selective small molecule inhibitor GSK-J4 induced cell death specifically in KDM6B/p16-addicted cell lines. This shows that the addiction of HPV-associated lesions and cancer to p16 expression creates a cellular vulnerability to KDM6B inhibition. De-repression of polycomb-silenced genes, such as p16, is a complex, multistep process. Hence, “epigenetic therapies” targeting KDM6B and potentially other enzymes that may also be rate limiting for maintaining the transcriptional competence of the p16 promoter may be efficacious in p16-addicted cancers.

It has been noted early on that p16 expression is confined to those tumors that contain pRB mutations, and a number of non HPV-associated cancer types, including some breast, prostate, lung and high-grade serous ovarian carcinomas express p16. It will be important to determine whether some of these tumors are similarly addicted to p16 expression and whether they are vulnerable to inhibition of KDM6B and/or other epigenetic enzymes that are necessary for p16 expression.

